# Charles R. Drew (1904-1950): A Pioneer of Blood Banking

**DOI:** 10.7759/cureus.76181

**Published:** 2024-12-22

**Authors:** Krishn B Patel, Kristal De La Cruz Quezada, Aishwarya Kalluri, Kenneth A Quezada, Donna Musegue, Carolina Rodriguez, Asha Plamoottil, Nadiya A Persaud, Latha Ganti

**Affiliations:** 1 Research, Orlando College of Osteopathic Medicine, Winter Garden, USA; 2 Research, Orlando College of Osteopathic Medicine, Orlando, USA; 3 Primary Care, Orlando College of Osteopathic Medicine, Winter Garden, USA; 4 Emergency Medicine and Neurology, University of Central Florida, Orlando, USA; 5 Medical Science, The Warren Alpert Medical School of Brown University, Providence, USA

**Keywords:** biographies, blood bank, historical vignettes, medical innovation, medical stories

## Abstract

Dr. Charles Richard Drew, a pioneering figure in modern blood banking and 20th-century medicine, revolutionized blood donation and storage processes, fundamentally shaping the field as we know it today. His extensive work with blood and plasma, combined with an innovative approach to reducing contamination, laid the foundation for modern standards in safety and efficiency. His efforts were instrumental in creating blood banks that supported soldiers during World War II. Beyond his scientific contributions, Dr. Drew was a tireless advocate for racial equality, challenging the widespread discrimination and segregation of his time, and paving the way for greater representation of Black physicians in the medical profession. This article is a historical vignette on Dr. Charles R. Drew, exploring his transformative impact on science, medicine, and social justice.

## Introduction and background

Early life

**Figure 1:**
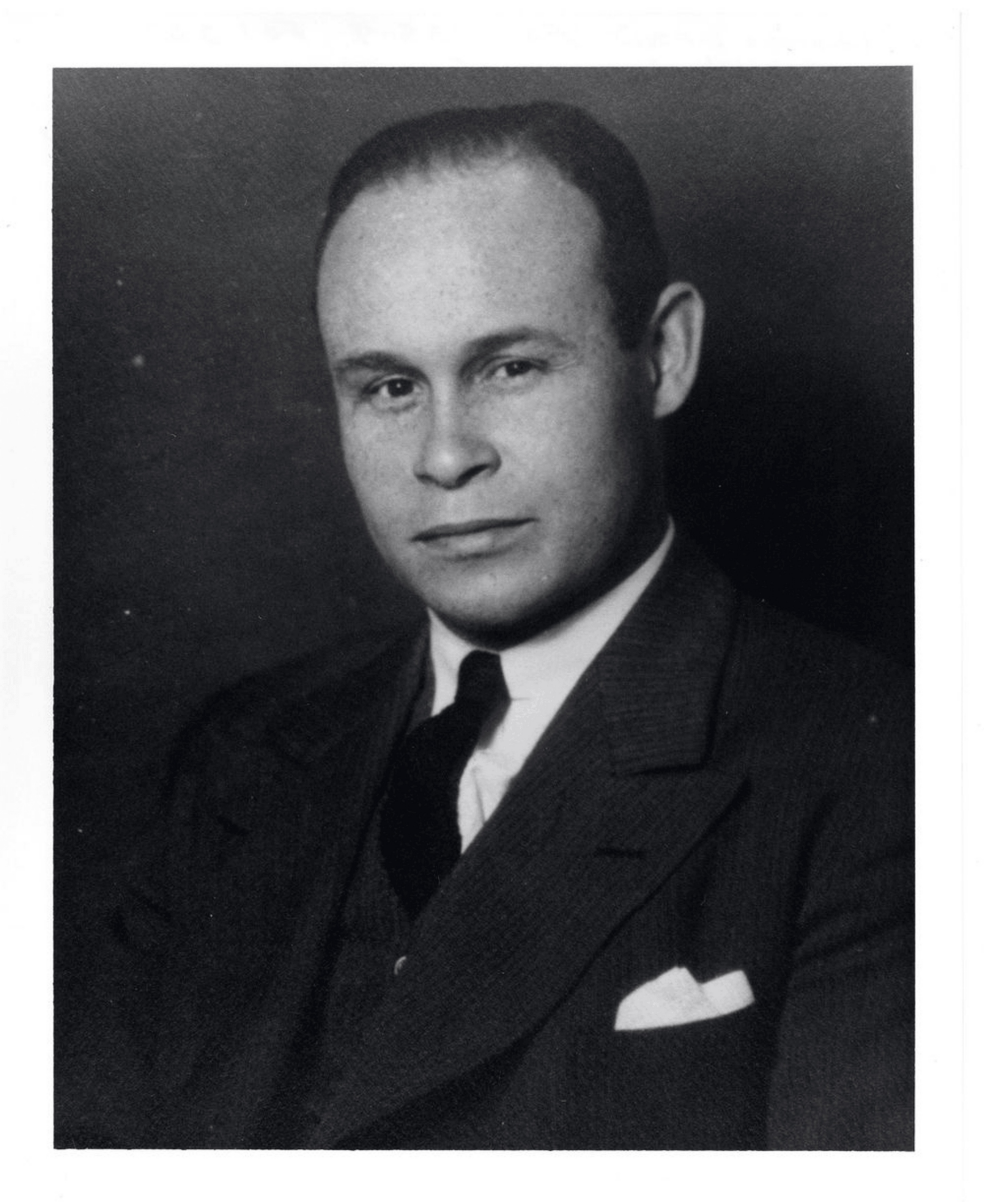
Portrait of Dr. Charles Richard Drew Image credit: [4]

Charles R. Drew (1904-1950) (Figure 1) was born on June 3, 1904, in Washington, D.C., to Richard Drew, a carpet layer, and Nora Burrell, a teacher, as the eldest of five children [1]. Drew was raised in a middle-class, interracial neighborhood where his parents emphasized the importance of academic excellence, civic responsibility, and faith [1]. Drew attended Dunbar High School, an elite all-Black institution, where he shined in athletics and was recognized as “one of the greatest high school athletes in this country” by the *Evening Star* newspaper in 1922 [1,2]. In 1922, he received a partial athletic scholarship to Amherst College in Massachusetts, where he excelled in football and track, ultimately graduating with an AB degree in 1926 [1]. As one of the few African American people at Amherst, Drew faced significant racial hostility, especially from opposing teams - a challenge that would continue to follow him into his professional life [3]. After graduating, Drew began teaching biology at Morgan State University in Baltimore, Maryland, where his passion for science and medicine continued to grow.

Medical education

Drew’s interest in medicine was sparked by two significant events: the loss of his sister Elsie to influenza in 1918 and his prolonged hospitalization following a football injury during college [1]. Throughout his recovery, he would roam the halls of the hospital in his wheelchair, observing procedures and developing a growing fascination with the field of medicine [1]. Due to racial segregation at the time, Drew had limited options when applying to medical school. Only a few medical schools accepted Black students, including Howard University College of Medicine, Meharry Medical College, and Harvard Medical School [1]. Drew applied to both Howard and Harvard University, but Howard denied his application due to missing humanities credits, while Harvard placed him on a waitlist [1]. Eager to embark on his medical studies, Drew instead chose to attend McGill University in Canada, an institution renowned for its racial inclusivity [1]. In 1928, Drew ventured to Canada, where he earned his Doctor of Medicine and Master of Surgery degrees in 1933, graduating second in his class [1,5]. During his time at McGill University, he received a scholarship prize in neuroanatomy, was inducted into the Alpha Omega Alpha medical honor society, and was awarded the J. Francis Williams Prize [3,6]. Drew’s impressive academic achievements established him as a distinguished protégé and pupil.

Medical training

Drew completed his surgical residency at Montreal Hospital from 1933 to 1935, collaborating with bacteriology professor John Beattie on fluid replacement treatments for shock [3]. Although he was passionate about this work and sought to further his training in transfusion therapy at the Mayo Clinic, racial discrimination prevented him from pursuing this opportunity. Instead, he joined Howard University College of Medicine as a pathology professor, eventually climbing the ladder to become a surgical instructor and chief surgical resident at Freedmen’s Hospital [3].

Doctoral studies

In 1938, while pursuing his doctorate at Columbia University, Drew was awarded the Rockefeller Fellowship to further his medical training at Presbyterian Hospital in New York under the mentorship of Dr. Allen Whipple [3,5]. Unlike his White peers, who gained experience in clinical and surgical settings, Drew was assigned to work under Dr. John Scudder, who had received grant funding to establish an experimental blood bank [3]. Together, they refined techniques for processing and preserving blood plasma, which is more stable than whole blood [3,5]. Drew developed a technique to dry and reconstitute plasma, prolonging its storage time [3]. This research laid the foundation for his doctoral thesis, *Banked Blood: Studies in Blood Preservation *(1939) [5], and in 1940, Drew became the first African American graduate to earn a doctorate from Columbia University [7,8]. Drew’s dissertation explored blood physiology, the stability of red blood cells, and the impact of potassium diffusion on stored blood. He found that red blood cells remained largely intact for up to 30 days under refrigeration, while hemoglobin levels and the ability to carry oxygen were preserved [8]. This foundational experience and publication sparked Dr. Charles R. Drew's passion for blood banking and plasma preservation.

## Review

Blood banking

Drew’s doctoral research examined blood chemistry, transfusion methods, and factors affecting blood storage, such as anticoagulants, preservatives, container design, and temperature [3]. Drew’s innovative methods of separating plasma from erythrocytes allowed plasma’s shelf life to be extended up to two months [3,7,9]. Plasma, a clear yellow liquid rich in proteins and electrolytes, serves as a versatile blood substitute for replacing fluids and treating shock [3]. Unlike whole blood, plasma has a longer shelf life, resists deterioration during transport, is compatible with any blood type, and can be administered in many ways [3,7]. Although plasma cannot transport oxygen like whole blood, it was essential during World War II for replenishing volume and clotting factors [3,5].

Blood for Britain (BFB) campaign

In 1940, Drew was appointed medical director of the BFB campaign due to his groundbreaking advancements in blood banking and preservation [5,10]. Drew, alongside Scudder and E.H.L. Corwin, pioneered methods to extract, preserve, and ship roughly 14,500 pints of lifesaving plasma to Britain, England [3,6,7,10]. Drew coordinated the collection and processing of blood plasma from several New York hospitals to supply lifesaving plasma for war casualties [7]. This was done by separating the plasma using centrifugation and sedimentation, adding merthiolate as an antibacterial, conducting bacterial tests, and diluting it with saline prior to being sealed and packed for transport [3]. The BFB campaign concluded in January 1941, having collected 14,556 blood donations and shipped over 5,000 liters of plasma saline solution to Britain, England, through the Red Cross [3].

Bloodmobiles and segregation

Following the success of the BFB campaign, the American Red Cross launched a pilot program in February 1941 to mass-produce dried plasma for military personnel, appointing Drew as its assistant director [3]. Drew introduced “bloodmobiles,” which were refrigerated mobile blood donation trucks that improved blood donation in public spaces [9]. This innovative initiative solidified his reputation as a pioneer and earned him the title “Father of the Blood Bank” [3,9]. Enraged by the program’s racist policy of segregating blood by race, Drew condemned it as unscientific and discriminatory, emphasizing that plasma is identical regardless of race [2,3]. Angered by this injustice, Drew resigned in 1942, though the policy remained in effect until 1950 [3].

Legacy

In October 1941, Drew returned to Howard University, where he served for nine years as Head of the Department of Surgery and Chief of Surgery at Freedmen’s Hospital [4]. He devoted himself to training African American surgeons and advocating against the exclusion of Black physicians in medical societies, specialty organizations, and the American Medical Association [1].

On April 1, 1950, Drew and three other Black surgeons were driving from Washington, D.C., to Tuskegee, Alabama, to attend a conference [5,11,12]. Early in the morning, in North Carolina, Drew reportedly fell asleep at the wheel, causing the car to crash into a tree [1,5]. While his passengers sustained only minor injuries, Drew was critically injured and taken to a segregated local hospital [5,10]. Recognized as a pioneer in blood banking, the medical staff worked tirelessly to save him, but unfortunately, their efforts were unsuccessful, and he tragically succumbed to his injuries [1,11,13]. Drew left behind his wife, Minnie, a former Spelman College professor, their four children, and a legacy of medical advancements in blood preservation that continue to save countless lives [5]. Drew’s innovative “bloodmobiles” continue to be reflected in modern medicine through mobile blood buses, such as OneBlood’s “Big Red Bus” [14].

## Conclusions

Dr. Charles R. Drew profoundly impacted medicine and healthcare through his groundbreaking innovations in blood banking, including the development of blood donation centers and bloodmobiles, which are still widely used today. His contributions helped to save countless lives during World War II, and his methods continue to influence modern blood preservation. Motivated by his own experiences, Drew was a passionate advocate for racial equality and fought for the inclusion of Black physicians. Dr. Charles R. Drew's groundbreaking advancements in blood banking and storage not only transformed medical practice but also underscored his unwavering commitment to social equity and advocacy in healthcare. His enduring legacy inspires perseverance in the face of adversity and solidarity with those confronting similar challenges, solidifying his status as a pioneer and role model in medicine.
